# High level expression of nicotinamide nucleoside kinase from *Saccharomyces cerevisiae* and its purification and immobilization by one-step method

**DOI:** 10.3389/fbioe.2023.1134152

**Published:** 2023-02-15

**Authors:** Jian-Ju He, Xin-Xin Liu, Ying Li, Zhe Wang, Hong-Ling Shi, Yun-Chao Kan, Lun-Guang Yao, Cun-Duo Tang

**Affiliations:** ^1^ Henan Provincial Key Laboratory of Funiu Mountain Insect Biology and Henan Provincal Engineering and Technology Center of Health Products for Livestock and Poultry, Nanyang Normal University, Nanyang, China; ^2^ College of Life Science and Agricultural Engineering, Nanyang Normal University, Nanyang, China

**Keywords:** nicotinamide nucleoside kinase, β -nicotinamide mononucleotide, expression, metal affinity tag immobilization, *Saccharomyces cerevisiae*

## Abstract

Nicotinamide riboside kinase (NRK) plays an important role in the synthesis of β -nicotinamide nucleotide (NMN). NMN is a key intermediate of NAD^+^ synthesis, and it actually contribute to the well-being of our health. In this study, gene mining technology was used to clone nicotinamide nucleoside kinase gene fragments from *S. cerevisiae*, and the *Sc*NRK1 was achieved a high level of soluble expression in E. coli BL21. Then, the reScNRK1 was immobilized by metal affinity label to optimize the enzyme performance. The results showed that the enzyme activity in the fermentation broth was 14.75 IU/mL, and the specific enzyme activity after purification was 2252.59 IU/mg. After immobilization, the optimum temperature of the immobilized enzyme was increased by 10°C compared with the free enzyme, and the temperature stability was improved with little change in pH. Moreover, the activity of the immobilized enzyme remained above 80% after four cycles of immobilized reScNRK1, which makes the enzyme more advantageous in the enzymatic synthesis of NMN.

## Graphical Abstract

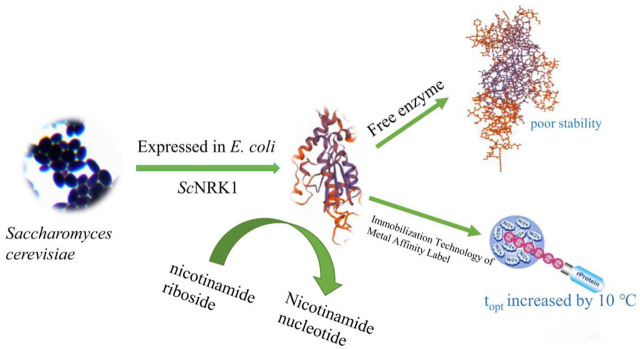

## Introduction

In NAD^+^ biosynthesis, β-nicotinamide mononucleotide (NMN) is a bioactive nucleotide formed by the reaction between phosphate groups and nucleoside-containing ribose and nicotinamide (NAMs) (Hong et al., 2020). NMN is a key intermediate of nicotinamide adenine dinucleotide (NAD^+^), an essential coenzyme for cellular redox reactions (Liu et al., 2021). Generally speaking, It has two forms, alpha and beta of which only beta is active with a molecular weight of 334.221 g/mol. NAD^+^ plays a crucial role in redox cellular balance, catabolism, and energy production and the NAD^+^ molecule acts as an electron carrier in all three realms of life (Hachisuka et al., 2018; Kalia, Shi Lee et al., 2019). In addition, NAD is a cosubstrate for enzymes that create signaling metabolites or posttranslationally modify proteins (Frederick et al., 2016). There have been many studies shown that NAD^+^ levels decline with age, in a wide range of species (Harrison et al., 2021). NMN needs to be transformed into NAD^+^ *in vivo* to perform its physiological function (Wang et al., 2016). Recently clinical studies have found that NMN administration reduces the risk of age-related diseases Alzheimer’s disease and type 2 diabetes (Longo and Kennedy, 2006; Wang et al., 2016). NMN has been called the “elixir” and some studies have shown that oral administration of NMN in the mouse model will lead to the recovery of NAD^+^, which can resist aging and prolong life (Das et al., 2019; Poddar et al., 2019).

NMN is a research hotspot in health products, food and cosmetics, and more and more attentions were paid to its synthesis. NAD^+^ is synthesized in mammalian cells through three different pathways: 1) synthesis of new tryptophan, 2) synthesis of nicotinamide or nicotinic acid, or 3) conversion of nicotinamide ribose nicotinamide ribose (NR) (Belenky et al., 2007). In some mammals, the rescue synthesis pathway takes NR as the substrate and phosphorylates to produce NMN under the action of nicotinamide nucleoside kinase (Yoshino et al., 2018). The synthesis of NMN begins with chemical synthesis. The most effective chemical synthesis method at present is the two-step method used in the 2017 Zhang’s group report: the β-isomer was eventually obtained in 85% yield after deprotection of the ester in methanolic ammonia (Zhang and Sauve, 2017). Owing to the chemical production of NMN is time-consuming and laborious, the biosynthesis of NMN has become a hot spot (Huang et al., 2022). Compared with traditional chemical catalysis, it has unique advantages such as mild conditions, low equipment requirements and strong stereoselectivity. According to the reported biotechnological methods of producing NMN in *Escherichia coli*, the highest NMN yield can reach 15.42 mg/L bacterial culture (or 17.26 mg/g protein) (Marinescu et al., 2018). It is reported that some people use whole cell catalysis to produce NMN. Extracellular production of 6.79 g/L NMN with glucose and NAM as substrates. The reaction selectivity from NAM to NMN is 86% (Shoji et al., 2021). Zhou Jingwen of Jiangnan University and others transformed *E. coli* into nicotinamide and glucose, creating a record high yield of NMN. The NMN titer reached 16.2 g/L, and the nicotinamide conversion rate was 97.0% (Huang et al., 2022). According to the current report, both were the highest. Compared with whole cell catalysis, fed batch fermentation with direct addition of nicotinamide is more conducive to the industrial production of NMN (Huang et al., 2022). Chemical synthesis using nicotinamide nucleoside (NR) as substrate depends on excessive phosphorus oxychloride and requires strict temperature operation which is not suitable for industrial (Qian et al., 2022). A new nicotinamide nucleoside kinase (*Klm*-NRK) from *Kluyveromyces martensii* and purified it. The specific activity of Klm-NRK was 7.9 U mg^−1^ protein, which was in the forefront of the reported NRK (Qian et al., 2022). Therefore, optimizing the enzymatic properties of nicotinamide nucleoside kinase plays an important role in the synthesis of NMN.

Immobilization technology is a mature and commonly used method in the development of enzymes. The main motivation of enzyme immobilization has always been to improve the performance of the enzyme. This can be achieved by improving the activity, stereoselectivity or stability of the enzyme. The enzyme exists in an appropriate physical form so that it can be recycled and reused at the end of biotransformation (Federsel et al., 2021). In this study, we plan to use gene mining technology to clone nicotinamide nucleoside kinase gene fragments from *Saccharomyces cerevisiae*, and then use pET28a plasmid to achieve a high level of soluble expression in *E. coli* BL21, and analyze its free enzymology characteristics, and then the nicotinamide nucleoside kinase was immobilized by metal affinity labeling to optimize the enzyme performance. The metal affinity label is hexahistidine (6 × His), which can gently bind to the C or N terminal of the enzyme molecule, and then specifically bind to Ni^2+^ on the carrier, so as to achieve the directional immobilization of the enzyme on the carrier (Wu and Filutowicz, 1999). The temperature stability of immobilized enzyme was improved, and the enzyme could be reused, simplifying the experimental process, which can effectively synthesize NMN.

## Materials and methods

### Reagents and kits

NR, NMN and Adenosine triphosphate (ATP) were purchased from Macklin (Shanghai, China). Commonly molecular biology reagents and kits, mainly including restriction enzymes, DL 2,000 DNA Marker and PrimeSTAR^®^ HS DNA Polymerase and so on, were purchased as previously reported (Wang et al., 2021). Methanolwere purchased from Komiou (Tianjin, China).

### Strains, plasmids, and culture media

Carrying prokaryotic expression plasmid *E. coli* BL21 (DE3)/pET28a*, S. cerevisiae* strains were preserved by our research group. The culture of *E. coli* uses LB medium, and YPD medium is used for the culture of S*accharomyces cerevisia*e (Tang et al., 2020a)*.*

### Web server and soft wares

National Center for Biotechnology Information (NCBI) was appled for searching nicotinamide nucleoside kinase sequence and structure. ProtParam tool was accustomed to predict theoretically physical and chemical parameters of target enzyme. DNAMAN was used for sequence analysis. TMHMM −2.0 was used to predict transmembrane region of the target enzyme. Origin 9.0 was used for data analysis.

### Cloning and expression of nicotinamide nucleoside kinase gene

With *S. cerevisiae* and nicotinamide nucleoside kinase as key words, the potential nicotinamide nucleoside kinase of *S. cerevisiae* was searched in NCBI. Based on the reported sequence, the primers *Sc*NRK1-F and *Sc*NRK1-R used to amplify nicotinamide nucleoside kinase1 (NRK1) gene sequence. According to this method (Tang et al., 2020b), Take the total RNA of *S. cerevisiae* as the template, and use PrimeScript ™ II 1st Strand cDNA Synthesis Kit is used to synthesize the first strand of cDNA, and then *Sc*NRK1-F and *Sc*NRK1-R primer PCR are used to amplify the coding gene of nicotinamide nucleoside kinase. The restriction endonucleases *Nde* Ⅰ and *Xho* Ⅰ are used to double digest the target gene and pET28a plasmid respectively, and then linked and transformed into *E. coli* BL21 (DE3) competent cells, obtained *E. Coli* BL21 (DE3)/pET28a *Sc*NRK1 recombinant strain.

### Expression and purification of nicotinamide nucleoside kinase1

The *E. coli* BL21 (DE3)/pET28a and recombinant *E. coli* strains containing nicotinamide nucleoside kinase encoding genes were picked and induced as described previously (Tang et al., 2019). Recombinant *E. coli* was induced by the reported method (Tang et al., 2018a). Then, we collected the induced recombinant cells through cryogenic centrifuge centrifugation, and The APV2000 high-pressure homogenizer (Gatesville, New York, United States) splits the cell lysate through high-pressure homogenization, centrifuges and purifies the collected cell lysate (Ma et al., 2009; Tang et al., 2018b; Hong et al., 2020). The protein concentration and sodium dodecyl sulfate-polyacrylamide gel electrophoresis of the nicotinamide nucleoside kinase1 were performed using reported previously method (Laemmli et al., 1970).

### Immobilized nicotinamide riboside kinase1

The cells collected by induction centrifugation were sonicated and then centrifuged to collect the supernatant, to which 2 mM MgCl_2_ and 1% NaCl were added to increase the binding amount of enzyme and agarose-Ni^2+^ column. The new column was equilibrated by washing with 5 times the column volume of binding solution, and the collected supernatant was added to the agarose-Ni^2+^ column and repeatedly suspended four times to increase the amount of enzyme binding, and finally the proteins were washed with deionized water (Jia et al., 2021). The agarose-Ni^2+^ column media was then aspirated, freeze-dried, and the drying was completed in a four-degree refrigerator for subsequent use.

### Enzyme activity and protein assays

NRK1 enzyme activity analysis: nicotinamide ribose (NR) was used as substrate, and NMN production was used as standard to calculate the enzyme activity. The determination of nicotinamide nucleoside kinase1 activity was slightly modified based on the reported method (Liu et al., 2021). The reaction system of free enzyme is 20 mmol/L pH5.8 Tris-HCL buffer, 10mmol/LMgCl_2_, 2 mmol/L ATP, 10 mmol/L NR, 0.1 mL diluted enzyme**.** The reaction system of immobilized enzyme is 20 mmol/L pH5.8 Tris-HCL buffer, 10 mmol/LMgCl_2_, 2 mmol/L ATP, 10 mmol/L NR, 0.03 g immobilized enzyme. Both were reacted at 40°C for 10 min, and the free enzyme was heated in boiling water for 5 min to terminate the reaction, and the reaction solution to be measured was filtered through a 0.22 μm filter membrane after the reaction was terminated. The immobilization reaction was terminated by centrifugation at 4°C and 12,000 rpm for 2 min. After centrifugation, the enzyme and liquid were separated. The supernatant was aspirated with a 1 mL syringe, the supernatant was filtered through a 0.22 μm filter membrane, and the precipitated immobilized enzyme was placed in −20°C for next use. Chromatographic conditions: Thermo Hypersil BDS C 18 column (250 mm × 4.6 mm, 5 µm), detection wavelength: 260 nm; injection volume: 10 μL. mobile phase: V (methanol): V (phosphate buffer) = 5:95 buffer; flow rate: 1 mL min^−1^. The amount of enzyme required to generate 1 μmol of NMN per minute under assay conditions was defined as 1 unit of enzyme activity. Then, use quantity one to estimate the apparent molecular weight of NRK1 (Tang et al., 2016).

### Temperature characteristics of the recombinant enzyme

According to the reported method, the temperature and pH characteristics of the determination of recombinant nicotinamide ribokinase1 were slightly modified (Hong et al., 2020). The optimum temperature of the free and immobilization recombinant nicotinamide nucleoside kinases1 was determined under the above standard determination conditions, except temperature ranging from 25 to 55°C. To estimate the thermostability, the free and immobilization recombinant nicotinamide nucleoside kinases1 were incubated at pH 5.8 and various temperatures (25–55°C) for 1.0 h, and then, the residual enzyme activity was measured under the optimal reaction pH and temperature.

### pH characteristics of the recombinant enzyme

The optimum pH of the free and immobilization recombinant nicotinamide nucleoside kinases1 was determined under the above standard determination conditions, except pH ranging from 3.4 to 8.8. To estimate the pH stability, the free and immobilization recombinant nicotinamide nucleoside kinases1 were incubated at various pH (3.4–8.8) for 1.0 h, and then, the residual enzyme activity was measured under the optimal reaction temperatures and pH.

### Kinetic parameters for the recombinant enzyme

The kinetic parameters of recombinant nicotinamide riboside kinase1 were determined with slight modifications according to the reported method (Tempel et al., 2007). To determine the specificity of NR substrates, the concentration of ATP in the immobilization system was 1 mmol/L. The catalytic activity of the recombinant enzyme was measured at optimal reaction condition with varied nicotinamide riboside concentrations from 0.01 to 0.03 mg. Each measurement was executed three times. Similarly, the specificity of ATP substrate was determined. The concentration of immobilized NR was 1 mmol/L, and the ATP concentration was set to 0.04–0.2 mg to determine the catalytic activity of the recombinant enzyme. Each measurement was executed three times. The apparent kinetic data for the enzyme exhibiting no substrate inhibition were calculated using the Michaelis-Menten equation (Ziegler et al., 2009). All calculations were performed by Origin 9.0.

### Reusability of reconstituted re*Sc*NRK1

To test the number of times the immobilized enzyme can be reused: the immobilized enzyme was used repeatedly to utilize the immobilized enzyme 10 times according to the above immobilized enzyme measurement method.

## Results and discussion

### Gene cloning and expression of nicotinamide riboside kinase1

**FIGURE 1:**
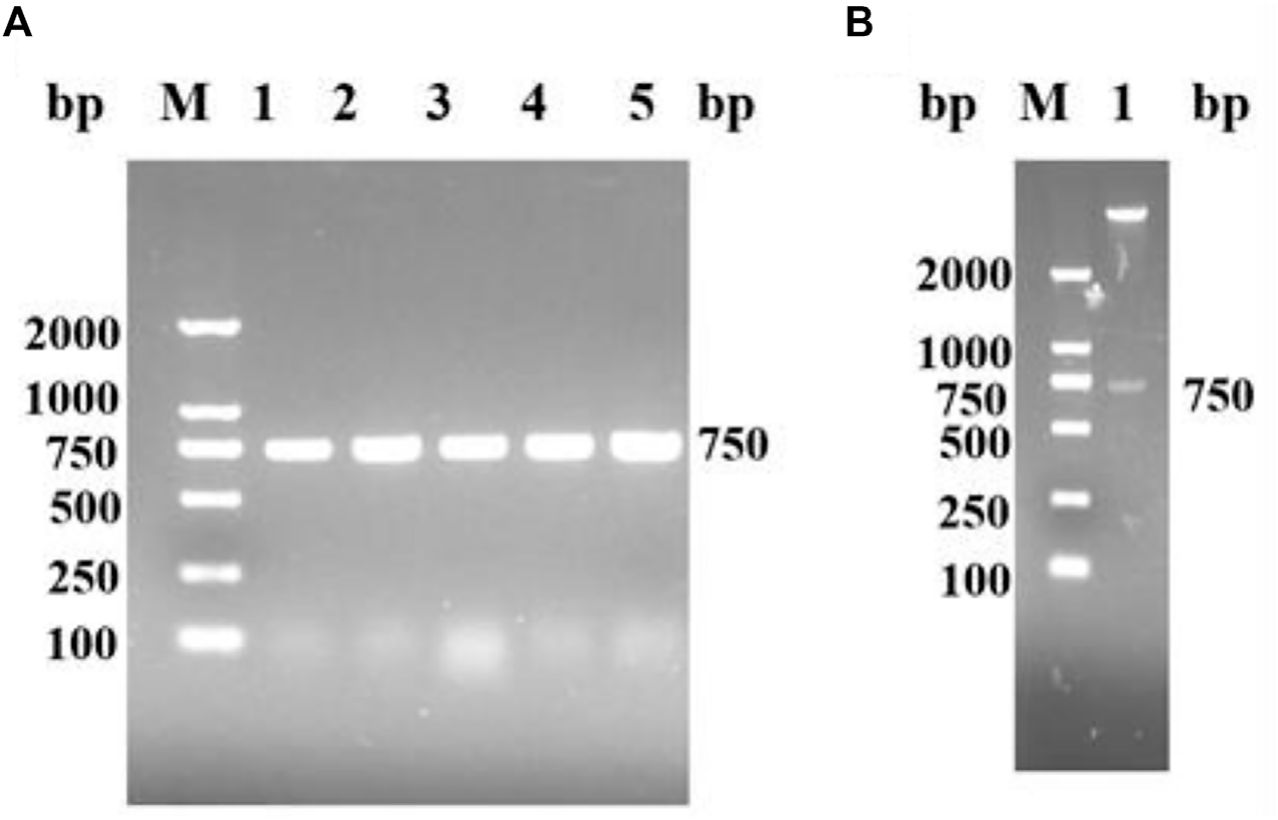
The agarose gel electrophoresis analysis for PCR products of *Sc*NRK1. and double enzyme digestion verification of recombinant *E. coli* BL21 (DE3)/pET28a -*Sc*NRK1. **(A)** PCR detection. Lane M, DNA marker; lane 1-5, PCR products from different strains of *E. coli* BL21 (DE3)/pET28a-*Sc*NRK1; **(B)** Double digestion detection. Lane M, DNA marker; lane 1, Double digestion products of *E. coli* BL21 (DE3)/pET28a-*Sc*NRK1 by *Nde Ⅰ* and *Xh* ***o*** *Ⅰ*.

A nicotinamide nucleoside kinase1 from *S. cerevisiae* was found in NCBI. The corresponding genome sequence (Genebank: AY611479.1) was designed to amplify the upstream and downstream primers of the target gene according to the corresponding gene sequence of *Sc*NRK1. *Sc*NRK1-F: GGA​ATT​CCA​TCA​TGA​TGA​TGA​CTT​CGA​AAA​AAA​AAA​AGT​GAT​A (including *Nde* I digestion site) and *Sc*NRK1-R: CCG​CTC​GAG​CTA​ATC​CTT​ACA​AGC​TTT​AG (including *Xho* I digestion site), Suzhou Hongxun Biotechnology was entrusted to conduct primer synthesis. The total RNA of *S. cerevisiae* was used as the template, and after reverse transcription, *Sc*NRK1-F and *Sc*NRK1-R were used as the primers for PCR amplification. The amplified products were subjected to agarose gel electrophoresis, as shown in Figure 1A, there were obvious specific bands at about 750 bp, and the length of the PCR product fragment was basically consistent with the expected theoretical length. The PCR products were purified and recovered with the kit, and then double digested with *Nde* Ⅰ and *Xho* Ⅰ. The digested products were connected to the pET28a plasmid which was also double digested, and transformed into *E. coli* BL21 (DE3) competent cells were screened for kanamycin resistance and detected by double enzyme digestion and double enzyme digestion electrophoresis as shown in Figure 1B. After identification, they were sent to Shanghai Sangon for sequencing and identification to obtain the gene sequence of *Sc*NRK1 and speculate its amino acid sequence.

### Sequence analysis of *Sc*NRK1

The deduced amino acid sequence of *Sc*NRK1 was predicted on ProtParam for theoretical physical and chemical properties. The results showed that the theoretical isoelectric point (*pI*) of *Sc*NRK1 was 6.23, the theoretical molecular weight was 27689.58 Da, and the instability index II was 25.59. *Sc*NRK1 was a relatively stable protein, and its half-life in *E. coli* and yeast could reach more than 10 h and 20 h respectively. The TMHMM—2.0 server was used to predict the transmembrane structure of the protein. The results showed that 240 amino acid residues of *Sc*NRK1 were extracellular residues, indicating that *Sc*NRK1 was theoretically easier to achieve extracellular secretory expression.

### Expression, purification and immobilizition of nicotinamide nucleoside kinase 1

**FIGURE 2:**
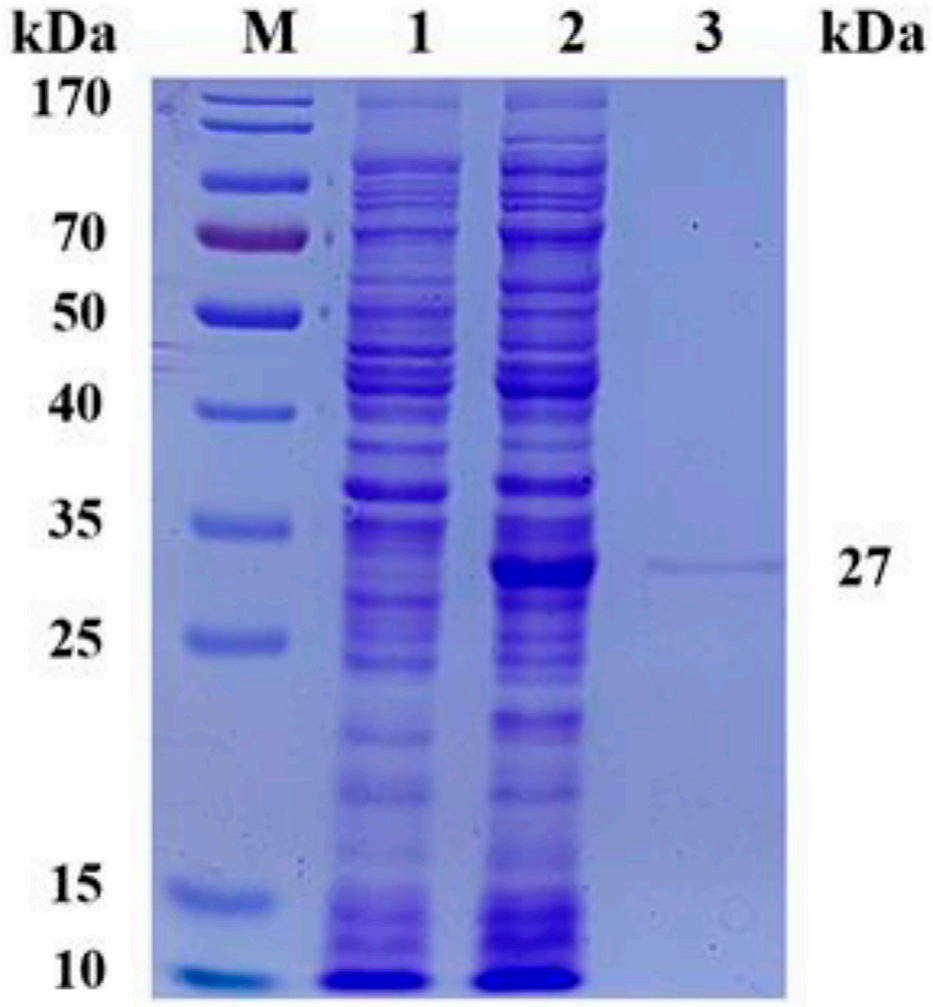
SDS-PAGE analysis of recombinant *Escherichia coli* expression products. SDS-PAGE analysis of recombinant *Escherichia coli* expression products M, PageRuler Prestained Protein Ladder; 1, expression products of *E. coli* BL21 (DE3)/pET28a; 2, crude expression product of *E. coli* BL21 (DE3)/pET28a-*Sc*NRK1; 3, purified expression product of *E. coli* BL21 (DE3)/pET28a-*Sc*NRK1.

To evaluate the expression level and enzymatic properties of *Sc*NRK1, the recombinant *E. coli* strain containing *Sc*NRK1 encoding gene was picked and incubated as above method. The re*Sc*NRK1 was purified by affinity chromatography with His tag, and the purified results were analyzed by SDS-PAGE (Figure 2). As shown in Figure 2, the re*Sc*NRK1 was successfully expressed in soluble form. Furthermore, the results indicated that the re*Sc*NRK1 was purified to homogeneity with apparent molecular weight of 27 kDa, which was consistent with its theoretical molecular weight of 27689.58 Da. After measurement and conversion, the dehydrogenase activity in fermentation liquor was 14.75 IU/mL. And the specific activity of the re*Sc*NRK1 toward nicotinamide riboside was 2252.59 IU/mg, which was significantly higher that previously reported (Bieganowski and Brenner, 2004), and had greater potential for application. The immobilized enzyme activity was 107.80 IU/mL, the activity of immobilized enzyme is 7 times higher than that of free enzyme. In this study, 10 mL of cell lysate was collected per 100 mL LB induction, and the lysate was passed through 2 mL agarose-Ni^2+^ column, and 0.523 g of immobilized enzyme was finally collected by freeze-drying. The recovery of enzyme activity of immobilized enzyme was 81%.

### HPLC analysis of NMN standard

**FIGURE 3:**
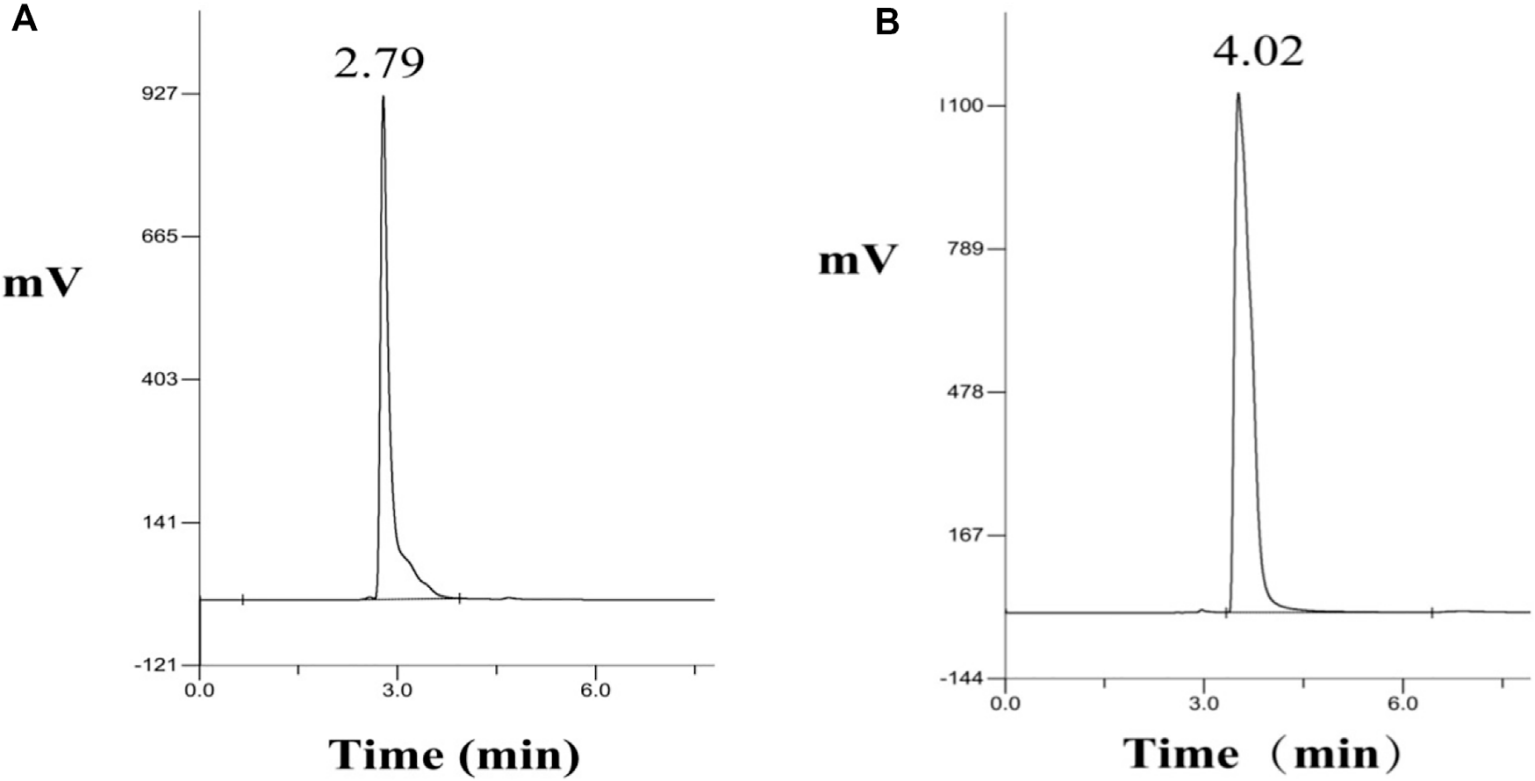
HPLC analysis of NMN standard and substrate NR. **(A)** Retention time of NMN standard in HPLC, **(B)** Retention time of NR standard in HPLC.

**FIGURE 4:**
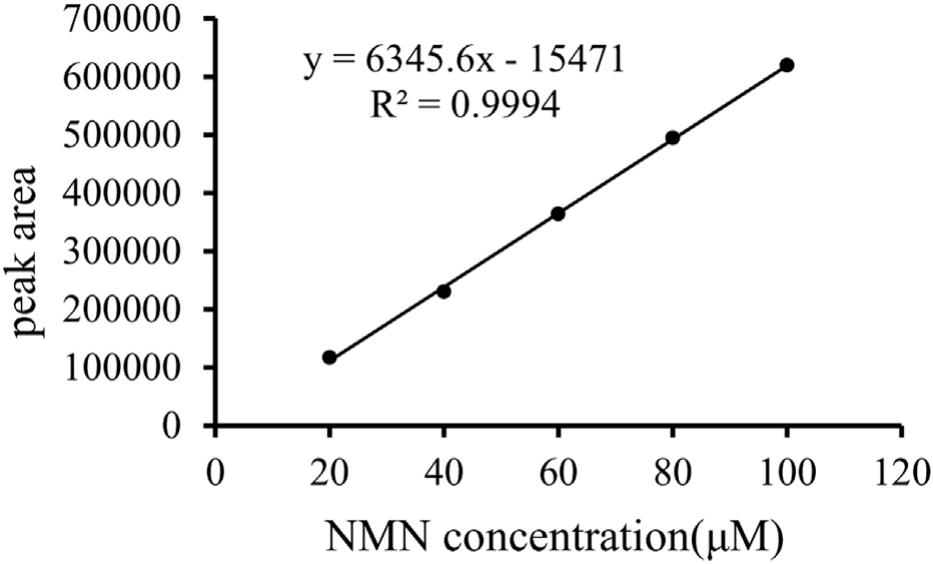
Standard curve of NMN. Prepare 100 μM NMN stock solution and dilute it to standard solutions of 20 μM, 40 μM, 60 μM, 80 μM and 100 μM respectively as standard koji of immobilized enzyme. The abscissa is the NMN standard concentration, and the ordinate is the peak area.

The analytically pure NMN was analyzed by HPLC on ThermoHypersil C 18 column. Under this analysis condition, the retention time of NMN was about 2.79 min, as shown in Figure 3A. The substrate standard was also analyzed by HPLC, with the retention time of 4.02 min, as shown in Figure 3B. As shown in Figure 4 the immobilized standard curve is y = 6345.6x—15471, R^2^ = 0.9994, The results show that under the HPLC analysis conditions and concentration range, the concentration of NMN is linearly related to the peak area. Therefore, this method can be used to accurately quantify NMN.

### Temperature characteristics of the re*Sc*NRK1

**FIGURE 5:**
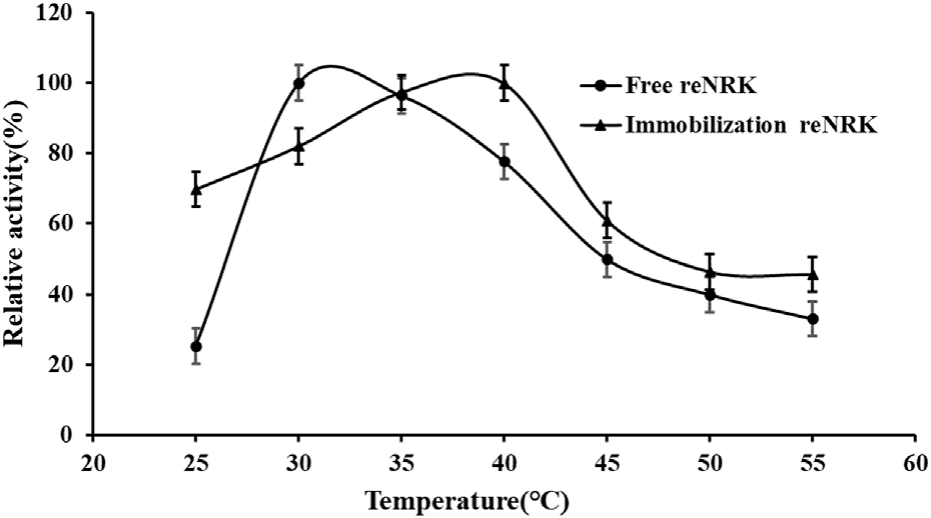
The optimal reaction temperature of the re*Sc*NRK1. The optimal reaction temperature of the re*Sc*NRK1 toward NR was determined under the standard assay conditions as above, except temperatures ranging from 25°C to 55°C.

**FIGURE 6:**
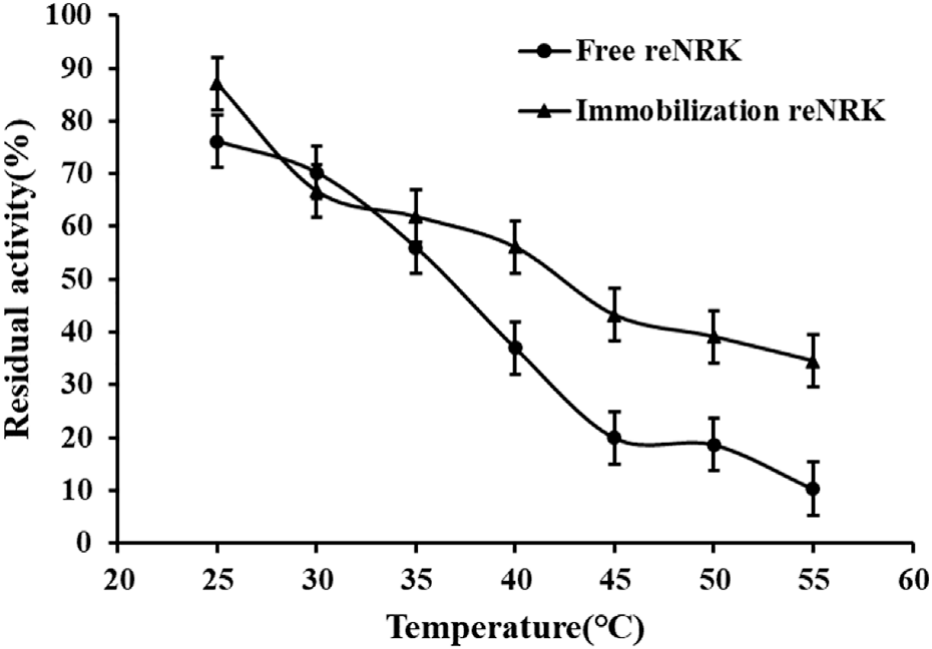
The temperature stability of the re*Sc*NRK1. The residual enzyme activity was measured at its optimal reaction temperature after being preincubated 1 h respectively.

The temperature characteristics of free and immobilization reScNRK1 were determined. The results are shown in Figure 5 and Figure 6 respectively. It can be seen from Figure 5 that when the free enzyme is in the temperature range of 20°C–30°C, the enzymatic reaction rate increases with the increase of temperature. When the temperature exceeds 30°C, the reaction rate decreases. The optimal reaction temperature of free enzyme is 30°C, which is not close to the physiological temperature of human body, but closer to the optimal growth temperature of bacteria. The optimum temperature of immobilization enzyme was 40°C, which was higher than that of free enzyme. It can be seen from Figure 6 that when the incubation temperature of free enzyme exceeds 40°C, its residual enzyme activity decreases significantly, only about 50%. The stability of the immobilized enzyme is relatively good, and the residual enzyme activity is 50% at 45°C. In general, the stability of the immobilized enzyme is improved. Metal affinity tag immobilization is a method to immobilize enzyme molecules on a carrier by forming coordination bonds between certain amino acids and transition metal ions (Fe^2+^, Co^2+^, Ni^2+^, etc.). The optimum temperature of immobilized enzymes is higher than that of free enzymes, which may be attributed to the decreased flexibility and increased rigidity of the enzyme after immobilization.

### pH characteristics of the re*Sc*NRK1

**FIGURE 7:**
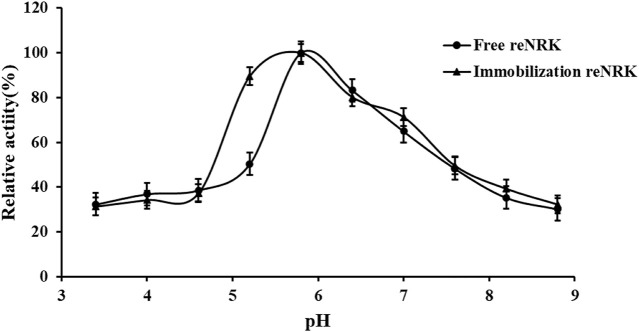
The pH optimum of the re*Sc*NRK1. The pH optimum of the r re*Sc*NRK1 was assayed by the standard activity assay method as stated above except the reaction pH values ranging from 3.4 to 8.8.

**FIGURE 8:**
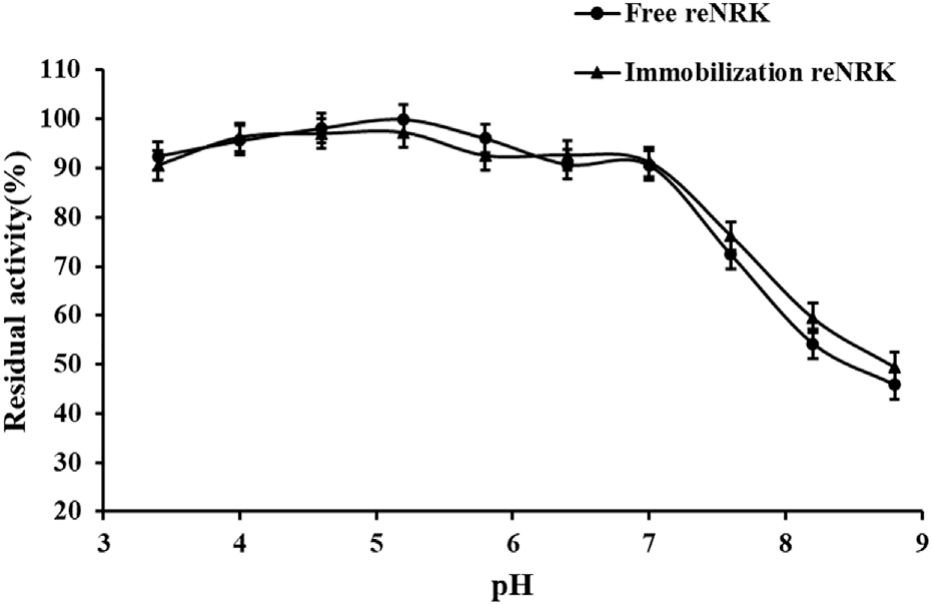
The pH stability of the re*Sc*NRK1. The residual activities were assayed under the optimal reaction temperatures and pH values after preincubating at 0°C for 1.0 h in varied pH values from 3.4 to 8.8.

The optimum reaction pH value and pH stability of free and immobilization reScNRK1 were determined. The pH of buffer too high or too low may lead to changes in spatial structure of the enzyme, thus reducing its catalytic activity (Shortall et al., 2021). The results are shown in Figure 7 and Figure 8. It can be seen from Figure 7 that re*Sc*NRK1 is an acidophilic enzyme, and its optimal reaction pH is 5.8. When the pH is far from its optimum pH and within the acidic range, its catalytic activity decreases slightly. However, when the pH value of the system is alkaline, the catalytic activity of the recombinant enzyme will decrease significantly. Therefore, it is necessary to control the reaction system to be acidic during use. It can be seen from Figure 8 that re*Sc*NRK1 has good tolerance to acidic pH, and its residual enzyme activity can be greater than 90% under measurement conditions, but its catalytic activity is significantly reduced under alkaline conditions.

### Kinetic parameters of the re*Sc*NRK1

In order to determine the specificity of NR substrate, modify the system NR and ATP concentrations according to the literature (Dölle and Ziegler, 2009). The kinetic parameters of the free and immobilized re*Sc*NRK1 toward nicotinamide riboside (NR) and ATP were determined according to the above method, and the results are shown in Table1. The free re*Sc*NRK1 exhibited high catalytic efficiency and affinity toward nicotinamide riboside, its *K*_m_ and *V*_max_ values were 18.69 mM and 98.39 μmol⋅min^−1^⋅mg^−1^. Meanwhile, it exhibited high catalytic efficiency and affinity toward ATP, its *K*_m_ and *V*_max_ values were 54.27 mM and 137.12 μmol⋅min^−1^⋅mg^−1^, indicating that the re*Sc*NRK1 has great potential in the rapid removal of nicotinamide riboside and ATP. Compared with the free re*Sc*NRK1, the *K*m value of the immobilized enzyme increased significantly, indicating that the spatial barrier after immobilization reduced the affinity of the enzyme to the substrate.

**TABLE 1:** Kinetic parameters of recombinant nicotinamide nucleoside kinase1.

Enzyme	Substrate	*K*_m_ (mM)	*V*_max_ (μmol⋅min^−1^⋅mg^−1^)
Immobilized enzyme	NR	54.76	46.97
	ATP	173.18	88.16
Free enzyme	NR	18.69	98.39
	ATP	54.27	137.12

### Reusability of reconstituted re*Sc*NRK1

**FIGURE 9:**
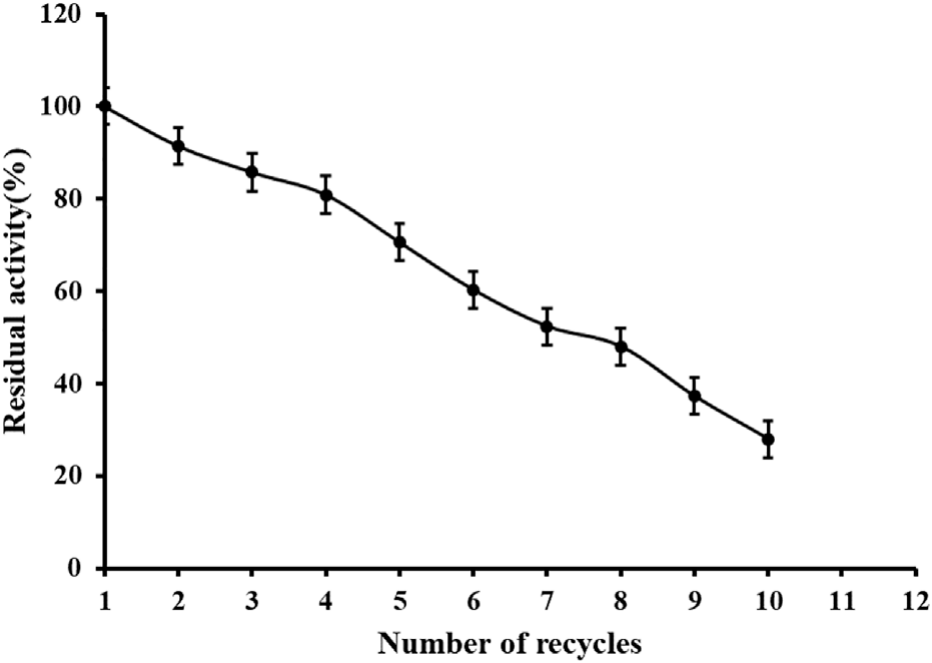
Reusability of immobilized re*Sc*NRK1. According to the above enzyme activity measurement method, repeatedly measure the activity of immobilized enzyme.

An important role of immobilized technology is that the enzyme can be reused. It can be seen from Figure 9 that the immobilized enzyme remains 80% active after 4 times of reuse, which is conducive to the recycling of enzyme and promotes industrial production. There are some disadvantages of metal affinity immobilization technology, such as tedious modification process of carriers and enzymes, and sometimes there are disadvantages such as easy dislodgement of metal ions, low selectivity and low affinity, which may be the reason of poor reusability. Factors affecting the adsorption and desorption of protein and Ni^2+^: 1) the adsorption and desorption of immobilized enzyme protein and metal Ni^2+^ are greatly influenced by the pH of the solution, usually the adsorption capacity is enhanced with the increase of the solution pH, because NRK1 is acidophilic enzyme and the reaction system is acidic, 2) the influence of ionic strength, at low salt concentration, the metal ions and protein mainly interact electrostatically, which will cause non-specific adsorption, but as the salt concentration increases, the positively and negatively charged salt ions cause a shielding effect between the protein and the metal ion. These two points may be the reason why the immobilized enzymes become less and less active in use.

## Conclusion

Nicotinamide mononucleotide is recognized as a nucleotide of NAD^+^ biosynthetic intermediate (Poddar et al., 2019), which is a research hotspot in food, cosmetics and health products. Genome mining technology can quickly transform hypothetical enzymes into real ones, providing more options for biocatalysis (Gong et al., 2013). In conclusion, in this study, nicotinamide ribokinase1 was successfully cloned from *S. cerevisiae* and solubilized for expression in *E. coli*, and the optimum temperature of the immobilized enzyme was increased and the temperature stability was improved. Immobilized nicotinamide riboside kinase1 can be recycled four times to maintain more than 80% activity, which implies a reduction in the cost contribution of the biocatalyst by reducing cost of the final product (Federsel et al., 2021). However, the number of times this can be recycled is small and the immobilization method will be improved subsequently.

## Data Availability

The raw data supporting the conclusion of this article will be made available by the authors, without undue reservation.
